# Challenges in the development of prognostic models utilising clinical trials data

**DOI:** 10.1186/1745-6215-14-S1-P115

**Published:** 2013-11-29

**Authors:** Kym Snell, Lucinda Billingham, Deborah Stocken, Richard Riley

**Affiliations:** 1 MRC Midland Hub for Trials Methodology Research, University of Birmingham, Birmingham, UK; 2 Cancer Research UK Clinical Trials Unit, University of Birmingham, Birmingham, UK; 3 Newcastle Clinical Trials Unit, Institute of Health and Society, Newcastle University, Newcastle, UK

## Aims

Prognostic models are statistical models that predict the risk of a future clinical outcome for individuals, and they are increasingly being developed using existing data from clinical trials. In this presentation we consider the challenges of this approach.

## Methods

Using data from two clinical trials in advanced stage pancreatic cancer, a prognostic model was developed using Royston-Parmar (R-P) flexible parametric survival models. R-P models use restricted cubic splines to model the baseline hazard function for time-to-event data and thereby allows absolute risk to be predicted for individuals. This contrasts the Cox regression approach which does not explicitly model the baseline hazard function.

## Results

We identified 6 key issues when using the trials' data to develop the prognostic model. These included dealing with multiple trials, handling multiple treatment groups with potentially different baseline hazard functions, and suitably using the data for model validation.

Informed judgement is needed to tackle these issues, and the baseline hazard function is pivotal to this. R-P models allow the baseline hazard to be displayed for all trials and all treatment groups. This aids decisions about whether to combine trials and treatment groups in the model development, and enables internal validation of predicted survival probabilities compared to observed probabilities from Kaplan-Meier plots.

## Conclusion

Clinical trials data is a rich source of existing data for prognostic model development, but it also provides many challenges. By modelling the baseline hazard, researchers can gain insight into how to tackle these issues.

